# Performance Comparison of Infection Prediction Scores in a South African Neonatal Unit: A Retrospective Case-Control Study

**DOI:** 10.3389/fped.2022.830510

**Published:** 2022-03-11

**Authors:** Lizel Georgi Lloyd, Angela Dramowski, Adrie Bekker, Nada Malou, Cecilia Ferreyra, Mirjam Maria Van Weissenbruch

**Affiliations:** ^1^Department of Pediatrics and Child Health, Faculty of Medicine and Health Sciences, Stellenbosch University, Cape Town, South Africa; ^2^Foundation for Innovative New Diagnostics, Geneva, Switzerland; ^3^Division IC Neonatology (NICU), Department of Pediatrics, VU University Medical Center, Amsterdam, Netherlands

**Keywords:** neonate, low birth weight, bloodstream infection, sepsis, infection prediction scores

## Abstract

**Background and objectives:**

Infection prediction scores are useful ancillary tests in determining the likelihood of neonatal hospital-acquired infection (HAI), particularly in very low birth weight (VLBW; <1,500 g) infants who are most vulnerable to HAI and have high antibiotic utilization rates. None of the existing infection prediction scores were developed for or evaluated in South African VLBW neonates.

**Methods:**

We identified existing infection prediction scores through literature searches and assessed each score for suitability and feasibility of use in resource-limited settings. Performance of suitable scores were compared using a retrospective dataset of VLBW infants (2016–2017) from a tertiary hospital neonatal unit in Cape Town, South Africa. Sensitivity, specificity, predictive values, and likelihood ratios were calculated for each score.

**Results:**

Eleven infection prediction scores were identified, but only five were suitable for use in resource-limited settings (NOSEP1, Singh, Rosenberg, and Bekhof scores). The five selected scores were evaluated using data from 841 episodes of HAI in 659 VLBW infants. The sensitivity for the scores ranged between 3% (NOSEP1 ≥14; proven and presumed infection), to a maximum of 74% (Singh score ≥1; proven infection). The specificity of these scores ranged from 31% (Singh score ≥1; proven and presumed infection) to 100% (NOSEP1 ≥11 and ≥14, NOSEP-NEW-1 ≥11; proven and presumed infection).

**Conclusion:**

Existing infection prediction scores did not achieve comparable predictive performance in South African VLBW infants and should therefore only be used as an adjunct to clinical judgment in antimicrobial decision making. Future studies should develop infection prediction scores that have high diagnostic accuracy and are feasible to implement in resource-limited neonatal units.

## Introduction

In 2015, neonatal infections contributed to over 500,000 deaths worldwide, the majority of which occurred in low- and middle-income countries (LMICs) (1, 2). Hospital-acquired infection (HAI; >72 h following birth) occurs 3–20 times more frequently in LMICs than in high-income countries and is a major contributor to neonatal morbidity and mortality (3). HAI among preterm (<37 weeks gestational age) and very low birth weight (VLBW; <1,500 g) infants is one of the most frequent complications encountered in neonatal units worldwide, affecting up to 20% of VLBW infants (blood culture positive episodes; proven HAI) with 28% experiencing multiple infection episodes (3). Reported mortality from proven HAI in Africa ranges from 27 to 72% (4–6), with up to 14–42% of surviving infants suffering significant cognitive impairment and 9–21% suffering from cerebral palsy secondary to central nervous system involvement, septic shock, and hypoxemia (7–9).

Despite HAI being common in neonates, the diagnosis thereof is challenging. Blood culture is considered the gold standard for the diagnosis of neonatal blood stream infections; however, the blood culture positivity rate is low and is affected by factors such as the volume of blood inoculated, the level of bacteremia and laboratory capability (10). Newer tests, such as interleukin-6, interleukin-8, procalcitonin, and real-time polymerase chain reaction (PCR) assays for bacterial detection (11, 12) have been investigated for applicability in neonates with HAI, but are not yet available in most resource-limited settings. For this reason, clinicians make use of additional tools such as infection prediction scores, i.e., combinations of clinical evaluations and readily available laboratory screening tests such as complete blood counts and C-reactive protein (CRP) tests, to guide decisions regarding empiric antimicrobial treatment initiation in neonates suspected to have HAI (13–15).

Although clinically useful, infection prediction scores have variable performance in different practice settings and should be validated locally before incorporation into routine use (16). When using infection prediction scores, clinicians should consider its diagnostic accuracy to avoid inappropriate or unnecessary antimicrobial therapy, which may promote development of antimicrobial resistance, compromise gastro-intestinal immunity, and contribute to adverse clinical outcomes (17, 18). There are several infection prediction scores available as screening tests for HAI in neonates. The first infection prediction score was developed by Tollner in Germany almost 40 years ago, using a combination of clinical evaluations and laboratory investigations (19). Subsequently, scores were developed in a variety of settings, including Belgium, Australia, India, Bangladesh, and Thailand, but none has been developed and validated in Africa. Diagnostic accuracy of these infection prediction scores varies widely across these existing models. In a meta-analysis of twelve infection prediction models (13), a NOSEP1 score of 8 or more by Mahieu et al. (20, 21) which made use of clinical and laboratory evaluations, showed the most potential for use, but authors cautioned that these models should be considered as guidance rather than an absolute indicator to commence or withhold antimicrobial treatment (13). Based on the vast differences in resources available in high-income countries compared to LMICs, scores which rely on indicators such as continuous heart rate monitoring, blood pressure monitoring, and laboratory testing may lack generalizability to LMIC settings.

The overall objective of this study was to evaluate the performance of neonatal infection prediction scores at a large, resource-limited South African neonatal unit with a high proportion of VLBW infant deliveries.

## Materials and Methods

### Study Design

Stage 1 of the study included a literature search strategy to identify English language publications pertaining to neonatal infection prediction scores, searching PubMed, Medline, and EBSCO Host databases between 1970 and 2020 or since inception, with inclusion of publications identified in the gray literature. Existing infection prediction scores were evaluated for their suitability and feasibility of application in LMIC neonatal units by assessing the availability of each score variable within neonatal units in a low-resource setting.

In Stage 2, performance comparison of the selected scores deemed appropriate and feasible for use in LMIC settings was performed against an existing dataset of HAI episodes in VLBW infants from Tygerberg Hospital, Cape Town, South Africa. The dataset was a retrospectively collected REDCap dataset of VLBW infants >72 h of age admitted between 1 January 2016–31 December 2017 (22, 23) which had been collected for an epidemiological study (publication under review). Since VLBW infants frequently have a hospital stay in excess of the 28-day neonatal period, any VLBW infants investigated for HAI during their neonatal unit stay were included. Medical records including demographic data, risk factors for HAI, relevant laboratory data, and clinical signs and symptoms on the day of investigation for suspected HAI were reviewed.

### Study Definitions and Population

Hospital-acquired infection episodes occurring after 72 h of admission to the neonatal unit were classified into three categories (24):

1. Proven HAI: Positive blood culture. Organisms were classified using the United States Centers for Disease Control (US CDC) list of pathogens and contaminants (25). Repeat blood cultures isolating the same pathogen within 10 days of the original specimen were considered to represent a single episode of infection. Patients who isolated coagulase-negative staphylococci (CoNS) from two separate blood cultures taken 24–48 h apart, or from a single positive blood culture combined with a serum CRP ≥10 mg/L and clinical features suggestive of infection, were included in the analysis. Contaminants were excluded from further analysis.
2. Presumed HAI: Clinical signs and symptoms of infection, such as respiratory distress, apnea, tachycardia, abdominal distention, temperature instability, lethargy, and vomiting; in the presence of a CRP ≥10 mg/L and a negative blood culture, where antibiotic treatment was continued for ≥5 days.
3. Excluded HAI: This included patients with short-term symptoms but no objective findings of infection, with negative blood culture, CRP <10 mg/L, where antibiotics were discontinued within 48–72 h based on local treatment guidelines.

Positive cultures from other sterile sites, e.g., cerebrospinal fluid and urine, and viral infections were not included in the analysis as the relevant data was not available.

The diagnosis of bronchopulmonary dysplasia was based on the Vermont Oxford Network algorithm of supplemental oxygen requirement at 36 weeks postmenstrual age (26). Patent ductus arteriosus was diagnosed according to the Vermont Oxford Network definition which incorporates a combination of Doppler echocardiogram and clinical criteria (26). Severe intraventricular hemorrhage was defined as grades III and IV hemorrhage according to the grading method described by Papile et al. (27).

Tygerberg Hospital is a 1,384-bed public teaching hospital in the Western Cape, South Africa. The obstetric-neonatal service manages approximately 8,000 high-risk deliveries (37% low birth weight; <2,500 g) and 3,000 neonatal admissions annually (28). The 132-bed neonatal unit includes a 12-bed neonatal intensive care unit (NICU), three high-dependency wards, and one kangaroo mother care ward.

### Statistical Analysis and Evaluation of Existing Scores

Statistical analysis was performed using IBM SPSS Statistics for Macintosh, Version 27.0 using an α level of 0.05. For normally distributed continuous variables means and standard deviations were calculated. Medians and interquartile ranges (IQR) were used for non-normally distributed continuous data.

Diagnostic test evaluation was performed using MedCalc Software, version 20.0.5. For each score the sensitivity, specificity, positive predictive value (PPV), negative predictive value (NPV), and likelihood ratios were calculated for proven HAI, and proven and presumed HAI, using the local dataset. Where specific variables in our VLBW infants were not available, it was replaced with a suitable related variable, e.g., pre-feed aspirates are not routinely performed in our setting; thus, this variable was replaced with vomiting in calculating the Singh score (29). Episodes where any of the variables were missing were not included in the analysis. Score cut-off values were based on those used in the original studies. A good screening test should have a low false-negative rate, and thus high sensitivity (30). A test with a positive likelihood ratio (PLR) of >10 or conversely a negative likelihood ratio (NLR) of <0.1, is considered a good screening test (30, 31). The discriminative performance of the scores were evaluated by assessing their receiver operating characteristic (ROC) curves and area under ROC curves (AUC).

All findings were reported in accordance with the STROBE-NI criteria (32). The Stellenbosch University Health Research Ethics Committee and the Tygerberg Hospital management reviewed and approved the study protocol (S20/11/325).

## Results

### Identification and Selection of Existing Neonatal Infection Prediction Scores for the Purpose of Performance Comparison

Eleven infection prediction scores were identified from the literature search (Table 1). Five of these scores [Tollner (19), Okascharoen et al. (33), Rubarth (34), Walker et al. (35), and Husada et al. (36)] were evaluated as unsuitable for use in resource-limited setting such as our South African unit as such as blood gas analysis, blood pressure monitoring, and continuous heart rate monitoring were not routinely performed on all patients. The Rodwell et al. hematological score (37) was also excluded as it uses seven hematological parameters, of which four require differential white blood cell counts. Although the authors stated that one or more parameters may be omitted without affecting the outcome of the score, the unreliability of using the differential count to diagnose infections was demonstrated by Schelonka et al. (38).

**TABLE 1:** Description of existing infection prediction scores for hospital-acquired infection in neonates.

Study (country)	Description of study population	Number of episodes used for validation (*n*)	Clinical parameters	Laboratory or procedure parameters	Score interpretation	Suitability for LMIC	Comment
Tollner (19) (Germany)	Any new-born 970–3,720 g 30–43 weeks	39	Skin color Microcirculation Muscle tone Bradycardia Apnea Respiratory distress Hepatomegaly Gastrointestinal symptoms	Metabolic acidosis (pH) Increased/decreased white cell count Left shift on differential white cell count Thrombocytopenia <100,000/mm^3^	0–4.5: no sepsis 0.5–10.0: observe >10.0: suspected sepsis	No	Score developed prior to introduction of antenatal steroids and surfactant Blood gasses are not routinely performed in many LMIC units
Rodwell et al. (37) (Australia)	Term and preterm 1–30 days of age	27	None	Immature to total neutrophil ratio (I:T) Total polymorphonuclear (PMN) leukocyte count Immature to mature neutrophil ratio (I:M) Immature PMN count Total white blood cell count Degenerative changes in PMNs Platelet count ≤150,000/mm^3^	≥3: sepsis	No	I:T and I:M ratios are not routinely performed in LMIC units
Mahieu et al. (20) NOSEP1 (Belgium)	Any new-born	Original study:50	Fever >38.2°C	CRP ≥14 mg/L Neutrophil percentage >50% Platelets <150,000/mm^3^ TPN ≥14 days	≥8: sepsis	Yes	May be used in larger LMIC neonatal units where total parenteral nutrition is used
Mahieu et al. (21) NOSEP-NEW-1 (Belgium)	Any new-born	External validation: 62	Fever >38.2°C	CRP ≥30 mg/L Neutrophil percentage >63% Platelets <190,000/mm^3^ TPN ≥15 days	≥11: sepsis	Yes	May be used in larger LMIC neonatal units where total parenteral nutrition is used
Singh et al. (29) (India)	Preterm 90%	External validation: 105 (41); 220 (39)	Grunting Abdominal distention Increased pre-feed aspirates Tachycardia Hyperthermia Chest retractions Lethargy	None	Weighted score: ≥2 definite and/or probable sepsis	Yes	The exclusive use of clinical variables makes this score very useful in LMIC units
Okascharoen et al. (33) (Thailand)	≤34 weeks 69% ≤1,500 g 49%	External validation: 119 (42)	Hypotension Abnormal temperature Respiratory insufficiency	Neutrophil bandemia >1% Thrombocytopenia <150,000/mm^3^ Presence of umbilical venous catheter	0–3: low risk 4–6: intermediate risk ≥7: high risk	No	Blood pressure monitoring not routinely performed in many LMIC units
Rubarth (34) (United States)	Any new-born	62	Skin color Perfusion Muscle tone Responsiveness Respiratory distress Respiratory rate Temperature Apnea	Increased/decreased white cell count Immature to total neutrophil ratio (I:T) Thrombocytopenia <100,000/mm^3^ Metabolic acidosis (pH) Total polymorphonuclear (PMN) leukocyte count	≥10: sepsis	No	Blood gasses and I:T ratio are not routinely performed in many LMIC units Complicated and cumbersome to perform score
Rosenberg et al. (39) (Bangladesh)	Out born ≤33 weeks	105	Pallor Jaundice Lethargy Apnea Hepatomegaly	None	≥1 clinical sign	Yes	The exclusive use of clinical variables makes this score very useful in LMIC units
Bekhof et al. (40) (Netherlands)	<34 weeks >72 h	178	Increased respiratory support Pallor or gray skin color Capillary refill time >2 s Lethargy	Central venous catheter in preceding 24 h	Nomogram	Yes	May be used in larger LMIC neonatal units where Central venous lines are used
Walker et al. (35) (Canada)	Any new-born	8	Maximum heart rate	Blood glucose Neutrophil bandemia Total polymorphonuclear (PMN) leukocyte count	Web based algorithm^1^	No	Not all neonates are on continuous heart rate monitoring in LMIC units
Husada et al. (36) (Thailand)	Hospitalized neonates (term and preterm) 7–28 days of life	208	Poor feeding Abnormal heart rate Abnormal temperature Abnormal oxygen stats	Abnormal leukocytes according to age Abnormal pH	0–2: low risk 3–4: medium risk 5–6: high risk 7–14: very high risk	No	Blood gasses are not routinely performed in many LMIC units

*^1^Formula: Ln (Odds of Bloodstream Infection) = −25.459 + 0.752 (Maximum Blood Glucose; mmol/L) + 0.119 (Maximum Heart Rate; beats per minute) + 0.108 (% Bands) + 0.071 (Maximum Neutrophils; ×109/L).*

Only five of the scores listed in Table 1 were considered feasible and appropriate for resource-limited settings (NOSEP1, NOSEP-NEW1, Singh, Rosenberg, and Bekhof scores) (20, 21, 29, 39, 40) and were therefore included in the current analysis. The NOSEP1 score included three laboratory features (CRP, platelet count, and neutrophil percentage), one clinical feature (fever) and one intervention (total parenteral nutrition), all of which are available in our setting (20). A variation of the NOSEP1 score, the NOSEP-NEW1, included the same parameters as the NOSEP1 score but used different cut-off values for each individual parameter (21). The Singh and Rosenberg scores used only clinical features, making them particularly useful in a resource-limited setting (29, 39). The Bekhof score consisted of three clinical features and one intervention (presence of a central venous catheter in the last 24 h) (40).

### Description of the Study Population

**FIGURE 1:**
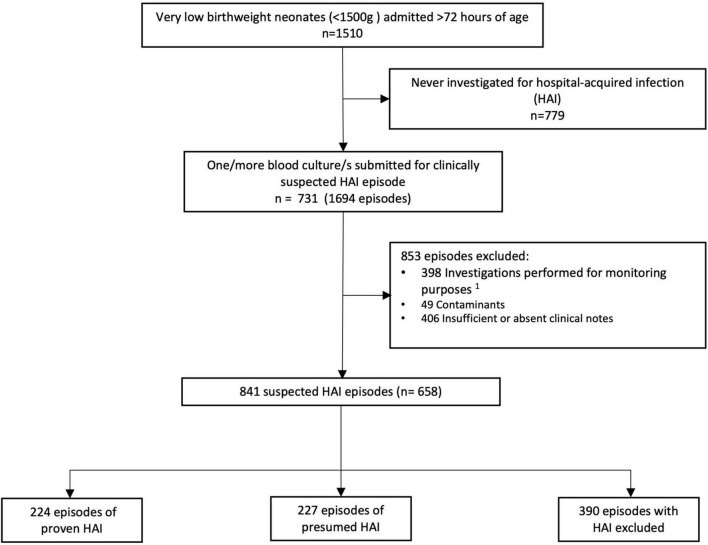
Flow diagram of neonatal hospital-acquired infection episodes in very low birth weight infants included in the analysis. ^1^Monitoring purposes: this refers to blood culture/s performed after 72 h of life in response to a positive blood culture or raised CRP obtained <72 h of life to monitor response to antimicrobial therapy.

Over a 2-year period, 1,510 VLBW neonates >72 h of age were admitted to the Tygerberg Hospital neonatal unit, of which 731 were investigated for 1,694 episodes of infection. Of these, 658 neonates with 841 episodes of HAI were eligible to be included in this study (Figure 1). Of the 841 suspected episodes of HAI investigated, 224 (26.6%) were proven HAI, 227 (27.0%) were presumed HAI and in 390 (46.4%) HAI was excluded.

The study population had a median birth weight and gestational age of 1,060 g and 28 weeks, respectively (Table 2). The majority of these infants were delivered *via* cesarean section and 10.3% were born outside of the tertiary center. Bronchopulmonary dysplasia, patent ductus arteriosus, and severe intraventricular hemorrhage were diagnosed in 18 (2.7%), 90 (13.7%), and 28 (4.2%), respectively. The overall mortality was 15.9%.

**TABLE 2:** Baseline characteristics of neonates included in this study (*n* = 658).

Variable	
**Demographic**	
Birth weight (g), median (IQR)	1,060 (900–1,226)
Gestational age at birth (weeks), median (IQR)	28 (27–30)
Small for gestational age, *n* (%)	89 (13.5)
Male gender, *n* (%)	312 (47.3)
Delivered by cesarean section, *n* (%)	421 (63.9)
Born outside of tertiary facility, *n* (%)	68 (10.3)
Born to mother living with HIV, *n* (%)	158 (24.0)
**Comorbidity, *n* (%)**	
Bronchopulmonary dysplasia	18 (2.7)
Patent ductus arteriosus	90 (13.7)
Severe IVH (grade 111 and IV)	28 (4.2)
**Risk factors present at time of infection, *n* (%)**	
Central venous catheter	83 (12.6)
Total parenteral nutrition	24 (3.6)
Outcome, *n* (%)	
Died	105 (15.9)

There was a Gram-negative predominance among the episodes of proven HAI (Table 3). At the time of investigation 83 (12.6%) had a central venous catheter *in situ*, and 24 (3.6%) were receiving total parenteral nutrition.

**TABLE 3:** Pathogen distribution in proven HAI group (224 episodes).

Organism	Number (%)
Gram-negative organisms	109 (48.7)
*Klebsiella* spp.	31 (13.8)
*Acinetobacter baumannii*	27 (12.1)
*Serratia marcescens*	25 (11.1)
*Escherichia coli*	11 (4.9)
Other^1^	15 (6.7)
Gram-positive organisms	76 (33.9)
*Staphylococcus aureus*	37 (16.5)
*Enterococcus* spp.	15 (6.7)
CoNS^2^	15 (6.7)
*Streptococcus agalactiae*	9 (4.0)
Fungi	4 (1.8)
*Candida albicans*	1 (0.4)
*Candida parapsilosis*	3 (1.3)
Polymicrobial	35 (15.6)
Total	224 (100.0)

*^1^Other: Enterobacter cloacae (n = 4) Pseudomonas aeruginosa (n = 3), Proteus mirabilis (n = 2), and unspecified other (n = 6).*

*^2^CoNS, coagulase-negative staphylococci.*

### Performance Comparison of Infection Prediction Scores (Table 4)

#### Proven Hospital-Acquired Infection

**FIGURE 2:**
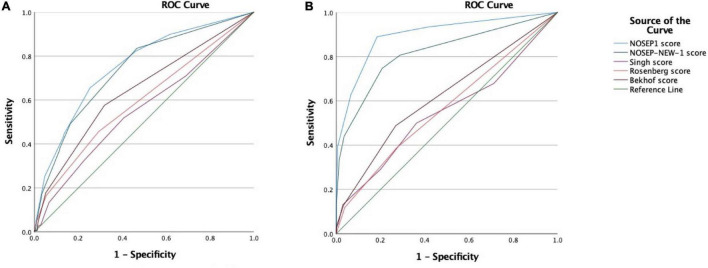
**(A)** Graph showing the receiver operating characteristic (ROC) curves for the analysis of the diagnostic accuracy of *the NOSEP1 score* (area under curve 0.753), *NOSEP-NEW-1 score* (area under curve 0.737), *Singh score* (area under curve 0.555), *Rosenberg score* (area under curve 0.594), and *Bekhof score* (area under curve 0.641), for the prediction of proven hospital-acquired infection. **(B)** Graph showing the ROC curves for the analysis of the diagnostic accuracy of the *NOSEP1 score* (area under curve 0.898), *NOSEP-NEW-1 score* (area under curve 0.820), *Singh score* (area under curve 0.550), *Rosenberg score* (area under curve 0.566), and *Bekhof score* (area under curve 0.620), for the prediction of proven hospital-acquired infection and/or presumed hospital-acquired infection. The *reference line* on both graphs represents a curve with no predictive value (area under curve = 0.50).

The highest sensitivity of 74% was achieved by the Singh score ≥1, paired with low specificity and PPV of 33 and 28%, respectively (Table 4). A NOSEP1 score ≥11 achieved a low sensitivity of 25%, specificity of 95%, NPV of 80%, and PLR of 5.37 and a NOSEP ≥14 achieved a sensitivity of 4%, specificity 99%, PPV 70%, and the highest PLR of 7.21. On the ROC curve analysis for the scores to compare the discriminative performance, the NOSEP1 and NOSEP-NEW1 achieved the highest AUC of 0.753 and 0.737, respectively (Figure 2).

**TABLE 4:** Performance comparison of previously reported infection prediction scores in very low birth weight infants at a South African neonatal unit, compared to the original studies.

			Sensitivity (%)		Specificity (%)		Positive predictive value (%)		Negative predictive value (%)		Positive likelihood ratio		Negative likelihood ratio	
						
Study	Model application	HAI	Original study	South African cohort	Original study	South African cohort	Original study	South African cohort	Original study	South African cohort	Original study	South African cohort	Original study	South African cohort
Mahieu et al. (20) NOSEP1	≥8	Proven	95	65	43	75	54	45	93	87	1.67	2.57	0.12	0.46
		Proven and presumed		62		94		91		69		9.65		0.41
	≥11	Proven	60	25	84	95	72	63	75	80	3.75	5.37	0.48	0.79
		Proven and presumed		19		100		100		53		–		0.81
	≥14	Proven	26	4	100	99	100	70	66	76	–	7.21	0.74	0.96
		Proven and presumed		3		100		100		49		–		0.97
Mahieu et al. (21) NOSEP-NEW1	≥11	Proven	84	17	42	97	64	61	32	78	1.45	4.9	0.38	0.86
		Proven and presumed		13		100		100		52		–		0.87
Singh et al. (29)^1^	≥1	Proven	87	74	29	33	38	28	85	78	1.2	1.1	0.44	0.8
		Proven and presumed	81	69	29	31	48	53	65	46	1.1	0.99	0.65	1.02
	≥2	Proven	53	56	80	59	52	33	81	79	2.65	1.36	0.59	0.75
		Proven and presumed	43	52	81	64	65	63	54	54	2.2	1.46	0.70	0.74
	≥3	Proven	13	32	90	76	36	33	72	75	1.3	1.32	0.96	0.90
		Proven and presumed	13	30	91	79	55	63	56	50	1.4	1.46	0.95	0.88
Rosenberg et al. (39)	≥1 clinical sign	Proven	77	46	50	72	65	37	65	79	1.54	1.64	0.46	0.75
		Proven and presumed		39		75		64		51		1.50		0.83
	≥2 clinical signs	Proven	42	17	82	95	73	54	54	76	2.33	3.29	0.71	0.87
		Proven and presumed		12		96		78		49		2.93		0.92
Bekhof et al. (40)	≥1 clinical sign	Proven	97	55	37	71	–	40	–	40	1.54	1.87	0.08	0.64
		Proven and presumed		46		75		69		69		1.88		0.71

*^1^Weighted variation of the score used.*

#### Proven and Presumed Hospital-Acquired Infection

A NOSEP1 score of ≥8 achieved a sensitivity of 62%, specificity of 94%, PPV of 91%, PLR of 9.65 and NLR of 0.41 (Table 4). The Singh score ≥1 achieved a higher sensitivity of 69%, and the NOSEP1 ≥11 and ≥14, as well as the NOSEP-NEW1 ≥11 all achieved specificities and PPVs of 100%. The highest AUC on the ROC curve analysis was achieved by the NOSEP1 and NOSEP-NEW1 of 0.898 and 0.820, respectively (Figure 2).

## Discussion

None of the five infection prediction scores we retrospectively evaluated, achieved sufficient diagnostic accuracy to recommend their routine use in our setting. To rule-out HAI, a score would have to achieve a high sensitivity (≥95% for a potentially lethal condition (16) and a NLR <0.1 (30, 31). None of the scores we evaluated can be used to rule-out HAI in our setting. To rule-in disease, a high specificity and PLR >10 is needed (30, 31). The NOSEP1 ≥8 achieved a specificity of 94% and PLR of 9.65 for proven and presumed infection and may be a useful adjunct to rule-in disease in our setting.

The strength of our study lies in the number of patients used to evaluate the infection prediction scores, far exceeding the sample size used in the original studies and including a previously unrepresented South African study population. The discrepancy found in the performance of these scores may be attributed to the fact that in this cohort we included only VLBW infants, admitted to a tertiary unit in a resource-limited setting. As VLBW infants have an increased risk of contracting a HAI during their hospital stay, we felt that they were the appropriate target group to use in this study. VLBW infants have a higher prevalence of respiratory symptoms due to prematurity, limiting the validity of any score that utilizes respiratory parameters.

A major limitation of our study was the inability to assess all the available infection prediction scores. This can be ascribed to the retrospective nature of the study, as well as our resource-limited setting. In our unit, the majority of the VLBW infants are cared for outside of the NICU in high- or intermediate care wards. Consequently, blood pressure and continuous heart rate monitoring data are not routinely available. Blood gas analysis is usually performed during a resuscitation and for ventilated patients but is seldom performed in patients investigated for HAI (Table 1).

The use of proven HAI, as well as proven and presumed HAI may be viewed as both a strength, and a limitation. Except for the Singh score, all the studies used a positive blood culture (proven HAI) as the reference standard, thus our comparison of proven HAI to combined proven and presumed HAI may provide valuable data to neonatal units where access to microbiological services may be limited. Four of the five studies reported a Gram-positive predominance amongst their positive cultures, compared to our Gram-negative predominance, and this may also have contributed to the different findings in our cohort.

Mahieu et al. developed and validated the NOSEP1 prediction score (20, 21) with one clinical, three laboratory, and one treatment variable, making its practical application in a resource-limited setting, where central venous catheters and total parenteral nutrition is often not available, difficult. The score performed well in ruling-in HAI in this South African cohort, with high specificity and high PLR. For the NOSEP1 score, Mahieu et al. (20) reported a sensitivity of 95% for a score of ≥8 for proven HAI, compared to our 65% for proven HAI, and 62% for proven and presumed infection. The Mahieu study included all neonates, with only 52% VLBW infants (54/80) in the study and had a Gram-positive pathogen predominance (82%) compared to the relative predominance of Gram-negative pathogens in our unit (48%). In a meta-analysis of prediction scores by Verstraete et al. (13), they concluded that the NOSEP1 score of greater than 8 had the greatest potential for use in the clinical setting. In our setting, it is likely most useful to rule-in HAI due to its high specificity and PLR. The NOSEP-NEW1 was developed as a variation on the NOSEP1 score and was found to improved performance in the external validation group in their study (21), however in our cohort it performed similar to the NOSEP1 score.

Singh et al. (29) developed a clinical score, specifically for resource-limited settings. Their score assessed for the presence of 7 clinical signs, regardless of gestational age. Kudawla et al. (41) performed an external validation in similar settings and achieved a sensitivity of 90% for a score ≥1, which compared well to the sensitivity of 87% that was achieved in the original study. In our cohort, the Singh score did achieve the highest sensitivity of all the scores assessed, but this was paired with the lowest specificity and PLR, which limits its usefulness in our setting. This difference is difficult to explain, as the Singh study cohort was very similar to ours, consisting of 91% preterm infants, and was performed in a tertiary level neonatal department in a resource-limited setting. The Singh score was also the only one that included proven and presumed infection in their analysis. However, in the Singh study the most common organism was *Staphylococcus aureus* (30%), at an incidence rate almost double that seen in our study (16%), which may partially explain the difference.

Rosenberg et al. (39) also failed to validate the Singh score in a cohort of premature infants and found that the inclusion of respiratory symptoms in a score that is used to assess premature infants who have a high incidence of underlying respiratory conditions unrelated to infection, was not appropriate. The Rosenberg score, with 5 clinical signs which did not include any respiratory parameters, achieved high specificity (95 and 96%) and moderately high PLR of 3.29 and 2.92 in the presence of ≥2 clinical signs in our cohort. In the original study they achieved higher sensitivity (42 vs 17% and 12% in our study), and a lower specificity. The cohort they used was similar (≤33-week gestational age infants) with a predominance of Gram-negative organisms of 87%, however they only used infants born outside of the referral facility compared to our cohort that had only 10.3% born outside of the referral facility. A recent review on impact of place of birth on outcomes of babies born between 1,000 and 1,500 g failed to demonstrate any difference, however, the review did not include any LMIC units, and further research is needed on this topic (43). The Rosenberg score has the potential to be used to rule-in disease in our setting.

The score developed by Bekhof et al. (40) incorporated only clinical signs, with one management parameter (central venous catheter for >24 h). Despite the patient cohort being similar in gestation (this study only included <34 weeks’ gestation premature babies), they achieved sensitivity and specificity of 97 and 37% in the original study, but we failed to replicate those results with a sensitivity of 55 and 46% and specificity of 71 and 75% in our cohort. As the Bekhof study was performed in a high-income setting with a Gram-positive HAI pathogen predominance, this may contribute to the difference in diagnostic accuracy when compared to our resource-limited setting. Neonates with Gram-negative HAI have a higher risk of adverse outcomes and death compared to Gram-positive HAI or no sepsis (44, 45).

The generalizability and feasibility of existing infection prediction scores in similar resource-limited neonatal units is an important consideration when diagnosing HAI in neonates and considering initiation of antibiotic treatment. An optimal infection prediction score should be user friendly, statistically sound, and generalizable to clinical settings outside of those in which the score was developed. External validation should be performed at another center or by different individuals (46).

## Conclusion

None of the five infection prediction scores evaluated in our study can be recommended for routine use in our setting. Future studies should develop bedside infection prediction scores using easily available clinical information and should assess the impact of infection scores on antibiotic prescribing behavior, until new low-cost and easy to use technologies can support HAI diagnosis.

## Data Availability Statement

The raw data supporting the conclusions of this article will be made available by the authors, without undue reservation.

## Ethics Statement

The studies involving human participants were reviewed and approved by the Stellenbosch University Health Research Ethics Committee. Written informed consent from the participants’ legal guardian/next of kin was not required to participate in this study in accordance with the national legislation and the institutional requirements.

## Author Contributions

LL, AD, AB, and MV conceptualized the study. LL collected and analyzed the data and prepared the first draft. All authors read, edited, and approved the final manuscript.

## Conflict of Interest

The authors declare that the research was conducted in the absence of any commercial or financial relationships that could be construed as a potential conflict of interest.

## Publisher’s Note

All claims expressed in this article are solely those of the authors and do not necessarily represent those of their affiliated organizations, or those of the publisher, the editors and the reviewers. Any product that may be evaluated in this article, or claim that may be made by its manufacturer, is not guaranteed or endorsed by the publisher.

## Abbreviations

CRP: C-reactive protein
HAI: hospital-acquired infection
LMIC: lower-middle income country
PLR: positive likelihood ratio
PPV: positive predictive value
NICU: neonatal intensive care unit
NLR: negative likelihood ratio
NPV: negative predictive value
VLBW: very low birth weight.
